# Dermoscopy and ultrasonography of Kaposi’s sarcoma nodules: new insights to guide intralesional chemotherapy?^☆^

**DOI:** 10.1016/j.abd.2021.03.017

**Published:** 2022-07-15

**Authors:** Gianluca Nazzaro, Athanasia Tourlaki, Carlo Alberto Maronese, Enrico Zelin, Emanuela Passoni, Lucia Brambilla

**Affiliations:** a Dermatology Unit, Fondazione IRCCS Ca' Granda Ospedale Maggiore Policlinico, Milan, Italy; b Department of Pathophysiology and Transplantation, Università degli Studi di Milano, Milan, Italy; c Dermatology & Venereology Department, Dermatology Clinic, Maggiore Hospital, University of Trieste, Trieste, Italy

Dear Editor,

Kaposi’s Sarcoma (KS) is a rare, Human Herpes Virus 8 (HHV-8) associated angioproliferative low-grade mesenchymal neoplasm, characterized by cutaneous patches, plaques, and nodules.^1^

Dermoscopy and Ultrasonography (US) are useful complementary techniques in the study of KS lesions,^2^, ^3^ the latter also providing valuable guidance for intralesional treatment.^4^ Correlation between dermoscopic, and ultrasonographic findings has not been reported in KS. Herein, we describe two cases of treatment-naïve, medium-to-large-sized KS nodules with complex architectural and vascular features, assessed by means of dermoscopy, and the US. We speculate that non-invasive recognition of complex KS lesional structure may aid in the adequate management of intralesional chemotherapy.

## Patient 1

**Figure 1:**
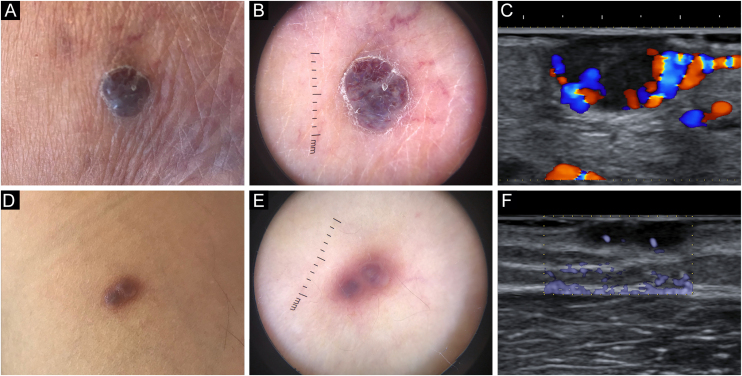
Clinical, dermoscopic (Dermlite DL200 Hybrid handheld device, 3 Gen, San Juan Capistrano, CA) and sonographic (ARIETTA 850 multifrequency 15.0‒18.0 MHz linear array transducer, Hitachi Medical Systems®, Zug, Switzerland) appearance of studied lesions from patients 1 (A‒C) and 2 (D‒F).

An eighty-two-year-old male with biopsy-proven, long-standing, classic KS and an otherwise unremarkable medical history complained of a newly formed lesion on the left heel, clinically appearing as a violaceous 9 × 6 mm nodule, with a peripheral scaly collarette. Dermoscopy showed two violaceus, large vascular areas separated by a white grayish structureless area (Fig. 1a‒b). At the B-mode examination, the lesion presented an oval hypoechoic structure with well-demarcated edges and with an inner median normoechoic septum delimitating two separate subunits. Color Doppler examination revealed that the subunits were supplied by two different blood vessels. Moreover, their blood flow did not communicate to a significant degree (Fig. 1c/Video 1 – supplementary material).

## Patient 2

A sixty-three-year-old male with biopsy-proven, long-standing, classic KS and an unremarkable history came in for consultation due to the appearance of an angiomatous 7 × 5 mm nodule on his right arm, presenting with a smooth surface and a faded border. Dermoscopy highlighted two violaceous structureless areas on a pinkish-brownish background, divided by a somewhat paler area laying in-between; moreover, no vascular structures could be appreciated (Fig. 1d‒e). On B-mode ultrasonography, the nodule presented two contiguous subunits and a septum-like structure could be noted in the center of the lesion. eFlow mode images confirmed the presence of distinct vascular peduncles supplying each subunit (Fig. 1f).

Adequate for size intralesional treatment with vincristine was offered in both cases, meaning the quantity of vincristine infiltrated was proportional to the largest diameter of the nodule, as measured clinically and dermoscopically.^5^ More specifically, 0.09 mL and 0.07 mL of vincristine sulfate (Vincristina Teva, Teva Italia Srl®, Assago, Italy) at a concentration of 1 mg/mL were administered in Patients 1 and 2, respectively.

Complete response was achieved in both cases, with no clinical evidence of recurrence in 12 months of follow-up.

Intralesional drug administration is particularly advantageous in nodular KS lesions, leveraging the presence of a pseudo-capsule for drug containment and concentration. Therapeutic failures and even paradoxical worsening in the days following the injection are rare but have been described. Known predisposing factors include large (7‒8 mm) lesional size and plantar and lateral plantar localization of the nodule.^5^ Septations delimiting autonomous vascular spaces within nodular KS lesions may theoretically lead to drug entrapment, relative over-filling, and subsequent inflammatory activation in surrounding tissues upon treatment. We presented two KS cases in which dermoscopy revealed whitish grayish structureless areas corresponding to septa upon ultrasonography. Further research is required to demonstrate a causal relationship between structural complexity and a proportion of the therapeutic failures observed with intralesional chemotherapy.

Although no definite recommendations can be given at this time, we argue that it would be cautious to screen nodular KS lesions for dermoscopic features suggestive of septations prior to intralesional treatment. Should any be noticed, a sonographic study as well as US-guided vincristine administration could be offered.^4^

## Financial support

None declared.

## Authors' contributions

Gianluca Nazzaro: Approval of the final version of the manuscript; elaboration and writing of the manuscript; obtaining, analyzing, and interpreting the data; intellectual participation in propaedeutic and/or therapeutic conduct of studied cases; critical review of the literature; critical review of the manuscript.

Athanasia Tourlaki: Approval of the final version of the manuscript; elaboration and writing of the manuscript; obtaining, analyzing, and interpreting the data; intellectual participation in propaedeutic and/or therapeutic conduct of studied cases; critical review of the literature; critical review of the manuscript.

Carlo Alberto Maronese: Approval of the final version of the manuscript; elaboration and writing of the manuscript; obtaining, analyzing, and interpreting the data; intellectual participation in propaedeutic and/or therapeutic conduct of studied cases; critical review of the literature; critical review of the manuscript.

Enrico Zelin: Approval of the final version of the manuscript; critical review of the manuscript.

Emanuela Passoni: Approval of the final version of the manuscript; intellectual participation in propaedeutic and/or therapeutic conduct of studied cases; critical review of the manuscript.

Lucia Brambilla: Approval of the final version of the manuscript; intellectual participation in propaedeutic and/or therapeutic conduct of studied cases; critical review of the manuscript.

## Conflicts of interest

None declared.
